# Implementing and tailoring a western-developed communication skills training program for graduate medical trainees in Qatar

**DOI:** 10.5116/ijme.5856.72b4

**Published:** 2017-01-14

**Authors:** Carma L. Bylund, Khalid Alyafei, Ambika Anand, Alanoud Al Marri, Walid Omer, Tripiti Sinha, Wahila Alam, Huda Abdelrahim, Abdullatif Al-Khal

**Affiliations:** 1 Department of Medical Education, Hamad Medical Corporation, Doha, Qatar; 2 Department of Pediatrics, Hamad Medical Corporation, Doha, Qatar; 3 Department of Surgery, Hamad Medical Corporation, Doha, Qatar; 4 Department of Dentistry, Hamad Medical Corporation, Doha, Qatar; 5 Department of Anesthesia, Hamad Medical Corporation, Doha, Qatar; 6 Department of Geriatrics, Hamad Medical Corporation, Doha, Qatar; 7 Center for Cultural Competence, Global and Public Health Division, Weill Cornell Medicine, Qatar, Doha, Qatar

**Keywords:** Communication skills training, residents, medical education, physician-patient communication, Qatar

## Introduction

In 2009, the Accreditation Council for Graduate Medical Education launched an international branch (ACGME-I). In July 2012, Hamad Medical Corporation (HMC) in Qatar was the second international organization to receive institutional accreditation. The evolution of the ACGME into an international organization leads to the need to understand how to effectively build upon educational programs developed in western cultures while still being attentive to important cultural differences and local needs of international programs.

Harden^1^ delineated three ways of approaching curriculum in international medical education: (1) local educators build a local curriculum; (2) a curriculum built in one country is exported to another; or (3) a transnational curriculum is developed with a strong international basis and attention to local students’ needs. All three approaches may be used by ACGME-I accredited institutions.

Communication skills training (CST) programs are of particular interest since the key concept of communication crosses many of the competencies and milestones. Yet communication, perhaps more than other competencies, is subject to differences in interpretation and cultural norms.^2^ For example, a communication competency such as “Create and sustain a relationship that is therapeutic for patients and supportive of their families”^3^ may be applied differently in international graduate medical education programs where the role of the family in healthcare is central.^2^

Important cultural differences notwithstanding, we began this project believing that there were more cross-cultural similarities than differences in healthcare communication skills. Thus, rather than develop a new program, we chose to tailor and implement a western model of CSTin graduate medical education in Qatar.Much has been published internationally on CST for medical students, graduate medical trainees and practicing physicians.^4^^-^^6^ Best practices for teaching communication skills are well-established,^7^ focusing on facilitator-guided, experiential work.^8^^,^^9^ Research on these programs generally show positive evaluations of such interventions and demonstrate skills uptake as measured with Standardized Patients (SPs). However, most published work about CST has been from western countries. Implementing a CST program in Qatar was innovative as we introduced a western-based curriculum. This is significant as previous educators have questioned whether an experiential role play approach would work in non-western countries.^10^ The primary purpose of this paper is to report on our experience tailoring and implementing a western-developed CST program in Qatar.

### Components of training

#### Setting

HMC is Qatar’s not-for-profit public healthcare system, consisting of eight public hospitals and other healthcare services, with 19 residency training programs, 14 of which are ACGME-I accredited. Residents and fellows are mandated to complete the CST course during their training.

#### Curriculum

Our CST course is based on the Comskil Model, developed by Memorial Sloan Kettering Cancer Center (MSKCC) in New York City.^5^ This theory-based model prioritizes the learning and application of concrete communication skills, within modules such as Breaking Bad News, Shared Decision Making, and Discussing Prognosis.

The implementation of the course follows best practice, including didactic sessions, exemplary videos, group discussions, and facilitator-led small and large group role plays with SPs. Our SPs are nurses, who received training on acting and giving feedback. Small group role play facilitators are trained faculty who took the CST and also completed a facilitator training session.^9^

Five HMC consultants attended a train-the-trainer course at MSKCC in November 2008, which began a partnership between the two institutions. MSKCC faculty provided train-the trainer courses at HMC, and the course was then piloted during subsequent years. In February 2014, based on feedback, we revised the course to its current form. Currently, trainees participate in seven modules over two separated, full days of CST, taken consecutively within one academic year.

#### Cultural Tailoring

The process of culturally tailoring the course has been ongoing, including changing the SP characters’ names and patient histories, revising didactic lectures, developing new videos, and developing multi-disciplinary role play scenarios. In addition, two of the original modules were changed substantially to allow for cultural differences. First, we modified the module on Conducting a Family Meeting to focus on the historically cultural practice of families asking doctors to withhold disclosure of life-threatening disease to patients. Second, the module on Working with Interpreters was changed to focus on working with untrained interpreters.

#### Feedback and Evaluation

During the time period of February 2014-June 2016, 345 participants completed both days of the workshop. Trainees reported high satisfaction with both days of the course, with the modules of Breaking Bad News and Shared Treatment Decision Making components rated as the most useful.  Trainees rated small and large group role play as the most helpful components of the workshops. About one-third of the participants reported that they had received CST during medical school. Those who did not have medical school CST gave higher ratings to some components of the course than those who did have medical school CST.

## Conclusions

Improving healthcare communication is an important task for graduate medical education, particularly in the Arabian Gulf region, where CST is uncommon, and where healthcare is being practiced in a strong multinational environment.^11^ Our experience demonstrates that implementing and tailoring a western-based CST for residents and fellows is feasible and acceptable in our ACGME-I accredited programs in Qatar. Whereas the majority of our residents did not receive communication skills training during medical school, those who did were less satisfied with parts of the course. Further, we found that Harden’s categories,^1^ as defined above, are not discrete. Instead, our experience demonstrates that it is possible to import a curriculum and then tailor it into a local curriculum.

We believe that our experience can serve as a model for other international medical educational programs that may bring in outside help to develop and initially implement a program, subsequently sustaining it using only local resources. Lessons learned from our experience include the following.

First, it is important to recognize that culturally tailoring a curriculum is a dynamic process. Although some simple modifications can be made initially (e.g., changing role play scenarios), it takes time to fully adapt. In addition to formal curricular changes, facilitators made small adjustments while teaching didactic and role plays each day as they recognized participants’ life experiences and cultural differences.  To effectively culturally tailor the curriculum, a standardized method of continual evaluation, feedback, and improvement was essential.

Second, significant effort was necessary to develop and maintain a quality group of instructors. In our institution, many of the faculty had never participated in CST, so it was necessary to have an accompanying approach of training physicians, first in communication skills, and second in facilitating small groups. Not only did this approach help build a strong cohort of course faculty, but was also critical to attend to the hidden curriculum.^12^ We have found that monthly newsletters recognizing faculty efforts, regular feedback, and booster courses are helpful in maintaining our more than 40 active instructors.

As the ACGME-I continues to expand, it is important to understand how best to share medical education ideas and programs across countries and cultures. This educational innovation successfully implemented and tailored a estern-developed CST program in an Arabian Gulf country and may serve as a model for other programs, especially around the important topic of physician-patient communication in such multicultural settings.

### Conflicts of Interest

The authors declare that they have no conflict of interest.
